# The Gut–Adipose–Tumor Axis in Obesity-Related Cancer

**DOI:** 10.3390/nu18081230

**Published:** 2026-04-14

**Authors:** Juan Feng, Yiyang Huang, Sien Lai, Tianhang Zhao, Yufen Xie, Xiangxing Zhu, Lian Liu, Dongsheng Tang, Aifen Yan

**Affiliations:** Guangdong Provincial Engineering and Technology Research Center for Gene Editing, School of Medicine, Foshan University, Foshan 528000, China

**Keywords:** obesity-related cancer, gut–adipose–tumor axis, gut microbiota dysbiosis, inflammation

## Abstract

The global obesity epidemic has emerged as a major driver of cancer incidence and mortality, with accumulating evidence highlighting the gut–adipose–tumor axis as a critical mediator of obesity-related carcinogenesis. The gut–adipose–tumor axis is a tripartite communication network, wherein the intestinal microbiome, adipose tissue, and tumor microenvironment engage in dynamic bidirectional crosstalk that alters cancer susceptibility and progression. This review synthesizes current understanding of the epidemiology, pathophysiology, therapeutic implications, and future directions of this axis. Obesity-induced gut dysbiosis leads to systemic dissemination of pro-inflammatory microbial products and metabolites. These gut-derived signals profoundly influence adipose tissue homeostasis, exacerbating chronic low-grade inflammation, promoting macrophage infiltration and polarization, and disrupting adipokine secretion patterns. Dysfunctional adipose tissue generates cancer-promoting mediators and metabolic perturbations. The convergence of gut-derived and adipose-derived signals creates a systemic pro-carcinogenic environment that reshapes the tumor microenvironment through multiple mechanisms. Understanding the gut–adipose–tumor axis as an integrated biological system offers opportunities for cancer prevention and treatment. This is of significant importance for exploring the mechanisms of obesity-related carcinogenesis and developing new therapeutic approaches for obesity-related cancers.

## 1. Introduction

In recent years, the incidence of obesity has increased significantly and is closely associated with the onset of various cancers. Obesity is not only a global public health issue but also considered one of the key environmental risk factors for many cancers. Evidence strongly indicates that overweight is linked to an increased risk of cancer, including endometrial cancer, esophageal carcinoma, pancreatic adenocarcinoma, gastric cancer, hepatocellular carcinoma, renal cell carcinoma, colorectal cancer, ovarian cancer, multiple myeloma, and thyroid cancer [1]. The global burden of obesity-related cancer has been further exacerbated by the COVID-19 pandemic, which has worsened obesity rates and disrupted cancer screening programs [2]. For example, the incidence of hepatocellular carcinoma (HCC) has shifted toward non-viral etiologies, with metabolic dysfunction-associated steatotic liver disease (MASLD) now the leading cause in developed countries, driven by obesity and alcohol use disorder [2,3]. The link between obesity and cancer is multifaceted, involving gut dysbiosis, metabolic dysregulation, chronic inflammation, and hormonal imbalances. These trends highlight the critical need to understand the mechanisms connecting obesity to cancer.

The gut–adipose–tumor axis is a complex, bidirectional network that integrates microbial, metabolic, and inflammatory signals to modulate cancer development. Gut dysbiosis, characterized by altered microbial composition and function, contributes to adipose tissue inflammation and metabolic dysfunction, which, in turn, creates a tumor-permissive microenvironment [4]. For example, in obesity, the gut microbiota shifts toward pro-inflammatory taxa (e.g., *Fusobacterium nucleatum*), which produce lipopolysaccharides (LPSs) that trigger systemic inflammation via toll-like receptor 4 (TLR4) signaling [4]. This inflammation promotes adipose tissue remodeling, with hypertrophic adipocytes secreting pro-inflammatory cytokines (e.g., TNF-α and IL-6) and adipokines (e.g., leptin) that drive tumor proliferation and angiogenesis [5]. Conversely, adipose tissue dysfunction can alter gut barrier integrity, leading to increased microbial translocation and further exacerbating inflammation [6]. Obesity-induced hyperinsulinemia activates insulin-like growth factor (IGF), promoting cell proliferation and inhibiting apoptosis in tumor cells [7]. Additionally, gut microbiota metabolize dietary components into carcinogenic metabolites (e.g., secondary bile acids), which directly damage DNA and promote epithelial–mesenchymal transition (EMT) [4]. For example, obesity-induced gut microbiota dysbiosis leads to excessive production of secondary bile acids such as deoxycholic acid (DCA) [8]. These metabolites directly damage DNA through oxidative stress and promote EMT in cancer progression [8,9]. Intestinal bacteria metabolize dietary choline and carnitine to trimethylamine (TMA), which is subsequently oxidized in the liver to trimethylamine N-oxide (TMAO) [10]. Elevated TMAO promotes inflammation, endothelial dysfunction, and has been linked to increased cancer risk [11,12]. Together, these interactions create a self-reinforcing cycle that drives obesity-related carcinogenesis, making the gut–adipose–tumor axis a key target for therapeutic intervention. Understanding the gut–adipose–tumor axis is critical for advancing cancer prevention, diagnosis, and treatment.

## 2. Gut Microbiota Dysbiosis in Obesity

**Figure 1:**
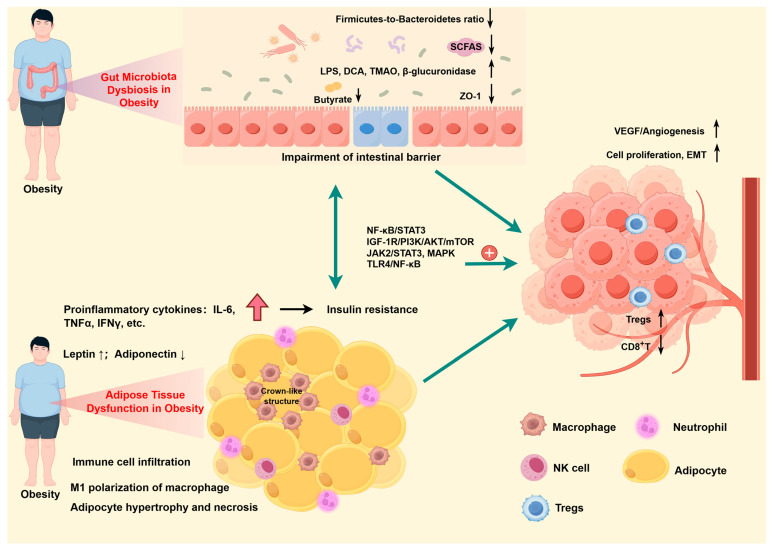
Pathophysiological mechanisms of the gut–adipose–tumor axis in obesity-related cancer. The main pathophysiological mechanisms of the gut–adipose–tumor axis in obesity-related cancer, including the gut microbiota dysbiosis in obesity, adipose tissue dysfunction in obesity, tumor microenvironment, and interactions among them. (By Figdraw 2.0). ↑: increased, ↓: reduced, +: promote.

The gut microbiota dysbiosis is closely related to the occurrence and development of obesity (Figure 1). Gut microbiota dysbiosis is often marked by a decrease in microbial diversity and the *Firmicutes-to-Bacteroidetes* ratio in obesity [13,14]. This dysbiosis contributes to metabolic endotoxemia, characterized by increased intestinal permeability and chronic low-grade inflammation, which further exacerbates obesity and its comorbidities, such as type 2 diabetes and cardiovascular diseases [15,16]. The presence of beneficial bacteria such as *Bacteroides thetaiotaomicron*, which is inversely correlated with serum glutamate levels, has been associated with reduced adiposity and improved metabolic profiles [17]. The depletion of beneficial species like *Methanobrevibacter smithii*, *Bacteroidetes*, *Lactobacillus plantarum*, *Lactobacillus paracasei*, and *Bifidobacterium animalis*, alongside an increase in *Fusobacteria*, *Proteobacteria*, and *Lactobacillus reuteri*, has been linked to obesity [18]. For example, the reduction in *Methanobrevibacter smithii* decreases overall fermentation efficiency and the production of beneficial short-chain fatty acids (SCFAs) [19]. *Bacteroidetes* depletion also reduces the production of SCFAs that regulate appetite and inhibit fat accumulation [20,21]. *Lactobacillus plantarum* and *Lactobacillus paracasei* contribute to intestinal barrier integrity, and their reduction leads to increased intestinal permeability and systemic endotoxemia [22]. *Bifidobacterium animalis* produces acetate and lactate, which acidify the gut lumen (inhibiting pathogens) and promote regulatory T cell differentiation. *Bifidobacterium animalis* depletion fosters immune dysregulation and systemic inflammation [23]. Conversely, the expansion of *Fusobacteria* and *Proteobacteria* promotes chronic low-grade inflammation and insulin resistance [24]. Collectively, these microbial shifts reduce energy expenditure, increase caloric extraction from diet, impair gut barrier integrity, and sustain the chronic inflammation that underlies obesity.

This microbial imbalance not only results in elevated levels of harmful substances but also correlates with reduced beneficial components such as short-chain fatty acids (SCFAs), adversely affecting intestinal health and metabolic function [15,25]. SCFAs such as butyrate can enhance calorie expenditure and energy metabolism by binding to G protein-coupled receptors, thereby modulating lipid metabolism and promoting the browning of white adipose tissue [26]. However, in obese individuals, SCFA production is often reduced, which is associated with decreased gut microbiota diversity and increased intestinal permeability [27]. Secondary bile acid, such as DCA, is markedly elevated under a high-fat diet and may lead to impairment of intestinal barrier function [28], which is also closely associated with alterations in the gut microbiota. Obese individuals harbor gut microbiota capable of extracting significantly more energy from their diet compared to lean individuals [29]. Gut microbiota influence enteroendocrine cells to modulate secretion of appetite-regulating hormones, including glucagon-like peptide-1 (GLP-1) and peptide YY (PYY) [30]. In obesity-associated dysbiosis, reduced production of GLP-1 and PYY impairs normal satiety signaling, promoting hyperphagia [31]. LPS derived from dysbiotic microbiota induces inflammation, which impairs leptin and insulin signaling and perpetuates obesity in a self-reinforcing cycle. These findings underscore the potential of targeting specific microbial species and their metabolic pathways as therapeutic strategies for obesity management.

## 3. Adipose Tissue Dysfunction in Obesity

Adipose tissue dysfunction is a pivotal factor in the pathogenesis of obesity-related metabolic diseases, including type 2 diabetes, cardiovascular diseases, MASLD, polycystic ovary syndrome (PCOS), chronic kidney disease, osteoarthritis, and a spectrum of obesity-related cancers. The dysfunction is characterized by a proinflammatory adipokine secretion and inflammatory cell infiltration (Figure 1), which is exacerbated by genetic, behavioral, and environmental factors leading to adipocyte hypertrophy, ectopic fat accumulation, hypoxia, and impaired mitochondrial function [32]. This inflammatory state is further accompanied by macrophage polarization mediated by mitochondrial dysfunction, which induces adipose tissue inflammation and systemic insulin resistance through the activation of the NLRP3 inflammasome and subsequent IL-1β release [33].

The storage capacity of subcutaneous adipose tissue (SAT) is limited, leading to ectopic fat accumulation and lipotoxicity, which in turn fosters local inflammation and insulin resistance [34]. This chronic low-grade inflammation is a hallmark of adipose tissue dysfunction and is closely related to metabolic disease, as dysfunctional adipocytes secrete inflammatory adipokines and attract bone marrow-derived immune cells [35]. Perivascular adipose tissue (PVAT) also plays a significant role in obesity-related vascular dysfunction. Under normal conditions, PVAT exerts anti-contractile effects through the release of vasorelaxants. However, PVAT contributes to vascular dysfunction by secreting vasoconstrictive and pro-inflammatory factors in obesity, leading to hypertension and endothelial dysfunction [36,37].

Adipose tissue inflammation further contributes to insulin resistance in obesity. The infiltration of pro-inflammatory macrophages and the secretion of cytokines such as TNF-α and IL-6 are critical in this process. Moreover, the role of hypoxia in adipose tissue dysfunction is significant, as it induces inflammatory responses and insulin resistance, further exacerbating metabolic dysfunction [38]. Metabolic changes such as insulin resistance, hyperglycemia, and adipokine dysregulation, like leptin and adiponectin, are closely related to adipose tissue inflammation in obesity [39].

## 4. Pathophysiological Mechanisms of the Gut–Adipose–Tumor Axis in Obesity-Related Cancer

### 4.1. Role of Gut Microbiota in Cancer Development

The gut microbiota, a complex ecosystem of microorganisms, influences host metabolism, immune responses, and inflammation, all of which are critical factors in the occurrence and development of obesity-related cancers. In a mouse model of liver cancer, antibiotic-mediated gut sterilization significantly reduces hepatic tumor burden, confirming the contribution of gut microbiota to carcinogenesis [40]. In colorectal cancer, gut dysbiosis in obesity is characterized by an increase in pro-carcinogenic taxa (e.g., *Fusobacterium nucleatum* and *Enterotoxigenic Bacteroides fragilis*) and a decrease in beneficial taxa (e.g., *Bifidobacterium* and *Lactobacillus*) [4]. For example, *Fusobacterium nucleatum* promotes colorectal cancer progression by activating TLR4 and NF-κB signaling, leading to increased IL-6 production and cancer cell proliferation [41,42]. In a mouse model, *Klebsiella aerogenes* colonization increases colonic tumor number and size, accompanied by elevated pro-inflammatory cytokines (TNF-α and IL-1β) [43]. Additionally, gut microbiota also produce carcinogenic metabolites. Obesity-induced changes in the gut microbiota promote the production of secondary bile acids (e.g., deoxycholic acid), which damage DNA and promote EMT [4]. Conversely, beneficial microbiota produce short-chain fatty acids (SCFAs) (e.g., butyrate), which inhibit histone deacetylases (HDACs) and induce apoptosis in tumor cells [44]. Emerging evidence indicates that dietary strategies aimed at modulating the gut microbiota, such as consuming dietary fiber or fermented foods to boost SCFA production, may contribute to a reduced risk of colorectal cancer [45,46,47]. Moreover, increased intestinal permeability allows translocation of LPS and other microbial products via the portal vein to the liver, activating hepatic TLR4/NF-κB signaling and promoting HCC progression in obese individuals [48]. *Fusobacterium nucleatum* and *Bacteroides fragilis* detected in pancreatic tumors are associated with worse outcomes, partly by suppressing anti-tumor immunity through toll-like receptor activation. Gut dysbiosis may seed the pancreatic microbiome via translocation, creating a pro-carcinogenic niche [49]. Gut dysbiosis in obese women alters circulating estrogen levels through β-glucuronidases that deconjugate estrogen metabolites, allowing their reabsorption and recirculation [50]. Elevated circulating estrogens constitute a major driver of postmenopausal breast cancer risk. These data demonstrate the critical role of gut microbiota in obesity-related cancer development.

### 4.2. Adipose Tissue Inflammation and Tumorigenesis

Adipose tissue inflammation is a key link between obesity and cancer, driven by hypertrophic adipocytes and immune cell infiltration. In obesity, adipocytes become hypertrophic and hypoxic, secreting pro-inflammatory cytokines (e.g., TNF-α, IL-6) and chemokines (e.g., MCP-1) that recruit macrophages [51]. These macrophages form crown-like structures (CLSs) around necrotic adipocytes, further amplifying chronic low-grade inflammation that underlies metabolic dysfunction and cancer risk [52,53]. In a mouse model of postmenopausal breast cancer, high-fat diet (HFD) feeding increases mammary adipose tissue inflammation, with CLS density correlating with increased tumor size [54]. Adipose tissue inflammation also promotes metabolic dysfunction. The resulting adipokine milieu establishes a systemic hormonal environment that directly stimulates oncogenic signaling, such as JAK2/STAT3 and NF-κB signaling [55]. Elevated leptin in obesity activates oncogenic signaling such as JAK2/STAT3, MAPK/ERK, and PI3K/AKT pathways [56]. TNF-α and IL-6 secreted by hypertrophic adipocytes and infiltrating macrophages activate NF-κB and STAT3 signaling in adjacent epithelial cells, which participate in immunosuppression, angiogenesis, and cell proliferation [57]. Hyperinsulinemia activates the IGF axis, and leptin promotes cell proliferation and inhibits apoptosis in tumor cells [39]. Additionally, adipose tissue-derived exosomes transfer microRNAs (e.g., miR-155) to tumor cells, promoting EMT and metastasis [58]. These mechanisms highlight the critical role of adipose tissue inflammation in tumorigenesis.

### 4.3. Interactions Between Gut Microbiota and Adipose Tissue

The gut microbiota and adipose tissue interact bidirectionally to modulate obesity-related cancer risk. The gut microbiota has been increasingly recognized for its influence on host metabolism and immune responses, which are critical in obesity and cancer. Obesity results in reduced diversity and dysfunction of the gut microbiota, which is closely associated with chronic low-grade inflammation and enhanced energy absorption [59,60]. This dysbiosis can also lead to an increased risk of various cancers by promoting inflammation and altering metabolic pathways [61]. The gut microbiota regulates cellular metabolism in the liver and adipose tissue by influencing the intestinal barrier and endocrine function, thereby affecting host lipid and glucose homeostasis as well as systemic inflammation [62]. *Fusobacterium nucleatum* produces an adhesin, which binds E-cadherin on epithelial cells and simultaneously disrupts the E-cadherin–ZO-1 complex that maintains tight junctions [63]. Butyrate deficiency in obesity creates an energy-deficient and pro-inflammatory colonocyte state that is prone to barrier disruption [64]. Adipose tissue dysfunction compromises gut barrier integrity by reducing the expression of tight junction proteins in intestinal epithelial cells, which increases microbial translocation [51]. Once barrier integrity is compromised, LPS enters the portal circulation, which further impairs barrier function, establishing a bidirectional self-reinforcing cycle. In an MMTV-PyMT transgenic breast cancer mouse model, HFD-induced gut dysbiosis accelerates mammary tumor onset and metastasis compared to lean controls [65]. Germ-free mice colonized with obese-donor microbiota gain more adipose mass compared to those colonized with lean-donor microbiota, directly demonstrating the causal role of gut microbiota in adipose tissue dysfunction [66].

Adipose tissue, particularly in obese individuals, is not merely a passive storage site for fat but an active endocrine organ that secretes various cytokines and adipokines, contributing to systemic inflammation. This inflammatory state is further influenced by gut microbiota-derived metabolites such as SCFAs and LPS, which can modulate immune responses and energy metabolism [67]. Moreover, the gut microbiota’s influence extends to the modulation of adipose tissue thermogenesis, a process that can counteract obesity by increasing energy expenditure. Certain gut bacteria can promote the browning of white adipose tissue, thereby increasing thermogenesis and reducing obesity [68]. This thermogenic effect is mediated through various pathways, including the activation of AMP-activated protein kinase (AMPK) and the modulation of bile acid metabolism, highlighting the complex interplay between gut microbiota and host metabolic pathways [69].

In conclusion, the gut microbiota and adipose tissue interact in a bidirectional manner to influence the pathogenesis of obesity-related cancer (Figure 1). This interaction is mediated through inflammatory pathways, metabolic regulation, and energy homeostasis, with the gut microbiota playing a central role in these processes (Table 1). Further research is needed to elucidate the precise mechanisms underlying these interactions.

## 5. Therapeutic Strategies Targeting the Gut–Adipose–Tumor Axis

### 5.1. Pharmacological Interventions in Obesity-Related Cancer

Pharmacological interventions in obesity-related cancer have garnered significant attention due to the intricate interplay between obesity and cancer pathogenesis. Pharmacological interventions targeting the gut–adipose–tumor axis include anti-obesity drugs, metabolic modifiers, and anti-inflammatory agents. Anti-obesity drugs such as GLP-1 agonists (e.g., liraglutide) are effective in reducing body weight and improving insulin resistance, as well as reducing the risk of specific types of obesity-related cancer in patients with type 2 diabetes (T2D) [70,71,72]. Beyond weight reduction, GLP-1 agonists can increase the abundance of *Akkermansia muciniphila*, improve intestinal barrier integrity, and reduce LPS translocation and systemic endotoxemia [73,74]. Another promising pharmacological approach involves the use of thiazolidinediones (TZDs) and metformin, which are traditionally used to manage insulin resistance in diabetes but have shown potential in cancer therapy. Metformin, a classic metabolic modifier and first-line therapy for T2D, inhibits the mTOR pathway and exhibits potential anti-cancer properties [75]. Metformin also increases the abundance of beneficial taxa such as *Akkermansia muciniphila* and *Bifidobacterium* [76], and reduces the production of inflammatory factors derived from adipocytes [77]. Metformin is linked to a reduced risk of colorectal cancer (adjusted RR = 0.884, 95%CI = 0.829–0.943) [78]. TZDs have demonstrated efficacy in reducing cancer cell proliferation and inflammation by regulating the PPARγ pathway [79]. Statins, widely used lipid-lowering and anti-inflammatory agents, also reduce the risk of breast cancer and colorectal cancer [80]. Statins increase anti-inflammatory taxa and reduce adipose tissue inflammation by inhibiting macrophage M1 polarization and reducing TNF-α/IL-6 secretion [81,82]. Other pharmacological agents include bile acid sequestrants, which reduce secondary bile acid levels and CRC risk [83]. Bile acid sequestrants primarily bind intraluminal bile acids, reducing secondary bile acid exposure of intestinal cells, and decreasing DCA-driven DNA damage and EMT signaling in the colorectal epithelium [84,85]. These pharmacological interventions offer promising strategies for cancer prevention and treatment in obese patients.

### 5.2. Lifestyle Modifications and Their Impact on the Axis

Lifestyle modifications, including diet and physical activity, are vital in influencing this axis. The modulation of gut microbiota through diet can significantly impact metabolic health and potentially alleviate obesity-related disorders, which are often precursors to tumorigenesis. A low-fat, high-fiber diet increases SCFA production, promoting gut barrier integrity and reducing inflammation [86,87]. Dietary interventions, such as a high-fiber diet, also enhance outcomes for patients undergoing cancer immunotherapy [88]. The modulation of gut microbiota through diet can significantly impact metabolic health and potentially mitigate obesity-related disorders, which are often precursors to tumorigenesis. For instance, flavones, a class of flavonoids, have shown promise in reducing obesity-induced inflammation and cancer risk by modulating gut microbiota composition and function [89]. Similarly, marine-algal-derived postbiotics have been identified as promising agents that can modulate the gut microbiota–adipose tissue axis, thereby reducing inflammation and improving metabolic outcomes [90].

Regular physical exercise reduces adipose tissue inflammation by inhibiting macrophage infiltration and pro-inflammatory cytokine production [91]. Aerobic exercise increases energy expenditure and modifies the profile of adipocytokines and myokines, which have paracrine and endocrine effects. These changes can lead to improved metabolic health and reduced inflammation, thereby potentially influencing cancer progression [92]. Additionally, exercise-induced modulation of adipose tissue can enhance the sensitivity of fibroblast growth factor 21 (FGF21), a key regulator of metabolic homeostasis. In obese individuals, exercise can reverse impairments in FGF21 signaling, thereby alleviating metabolic dysfunctions such as insulin resistance and ectopic lipid accumulation [93]. The role of exercise in modulating the inflammatory network extends to the hypothalamus, a critical brain region involved in energy homeostasis. Obesity induces hypothalamic inflammation and resistance to leptin and insulin, which are key hormones in energy regulation. Exercise has been shown to restore hypothalamic health by reducing inflammation and improving leptin sensitivity, which can improve metabolic disorders and obesity-related cancer [94]. There is strong evidence of a connection between physical activity and reduced risk of breast, colon, bladder, endometrial, esophageal adenocarcinoma, renal, and gastric cancers [95]. Physical activity can also decrease all-cause and cancer-specific mortality rates in patients diagnosed with colorectal, breast, or prostate cancer [95]. In addition, bariatric surgery has shown profound benefits beyond metabolic health, including a substantial reduction in cancer risk (odds ratio [OR] = 0.65; 95% CI = 0.53–0.80) [96]. These lifestyle modifications target multiple components of the gut–adipose–tumor axis, making them effective strategies for cancer prevention. These findings suggest lifestyle modifications could be effective strategies in managing obesity and preventing related cancers.

### 5.3. Emerging Therapies Targeting Gut Microbiota

Emerging therapies targeting gut microbiota include probiotics, prebiotics, synbiotics, and fecal microbiota transplantation (FMT). Probiotics, which are live microorganisms that confer health benefits to the host, can modulate gut flora and influence health through enhancing mucosal barrier integrity and immune modulation [97]. Probiotics also exert anti-tumor effects and enhance the efficacy of cancer therapies. They modulate the gut microbiota composition, improve intestinal barrier function, inhibit inflammation, and regulate immune responses, thereby potentially reducing cancer risk and improving treatment outcomes [98]. For example, in the prevention and treatment of colorectal cancer and breast cancer, probiotics exert an anticancer effect by altering the composition of the gut microbiota, inhibiting the activity of carcinogens, and enhancing the host’s immune response [99,100]. Probiotics such as *Lactobacillus rhamnosus* reduce pro-inflammatory taxa and increase beneficial SCFA production [101]. SCFAs possess anti-carcinogenic properties and may play a role in reducing obesity-related cancer risk [102]. In a mouse breast cancer model, *Lactobacillus reuteri* administration modulates the tumor immune microenvironment by downregulating immunosuppressive mediators (TGF-β, IL-10), suggesting enhanced anti-tumor immunity [103]. Prebiotics, non-digestible fibers that promote the growth of beneficial bacteria and metabolic products, also play a significant role in maintaining gut health and preventing dysbiosis, which is linked to cancer development. Prebiotics can increase the abundance of Firmicutes and reduce the abundance of Bacteroidetes, a microbial profile typically associated with a leaner phenotype [104]. The supplementation of galacto-oligosaccharides from lactulose significantly reduces the number of colonic tumors in a CRC animal model [105]. Inulin supplementation in a rat model significantly attenuates tumor development, reduces circulating pro-inflammatory cytokines, and increases colonic butyrate levels [106,107]. However, the effects of prebiotic interventions can vary among individuals, possibly due to differences in their genetic backgrounds. The combination of probiotics and prebiotics, known as synbiotics, offers a synergistic approach to modulating the gut microbiome, enhancing its diversity and functionality, which is particularly beneficial in managing obesity-related metabolic dysfunctions and cancer [108,109]. Synbiotic supplementation restores healthy gut microbiota composition and inhibits inflammation and tumor cell proliferation in CRC patients [110]. FMT, the transfer of fecal matter from a healthy donor to a recipient, aims to restore a balanced gut microbiota. It has shown promise in treating metabolic disorders such as obesity and type 2 diabetes, which are risk factors for cancer. FMT’s potential to remodel the gut microbiota and improve metabolic outcomes underscores its therapeutic value in obesity-related cancer [111,112]. FMT from healthy donors contributes to the normalization of gut microbiota and reduces tumor growth in a CRC mouse model [105]. These emerging therapies hold promise for targeting gut dysbiosis in obesity-related cancer. However, the clinical application of FMT in cancer therapy is still in its early stages, and ongoing researches are needed to optimize protocols and assess long-term safety and efficacy. Phase I/II trials have explored FMT from immunotherapy responders to melanoma patients refractory to PD-1 checkpoint inhibitors, reporting objective responses in 3/10 and 6/15 patients, respectively [113,114]. The variable efficacy reflects the polygenic, diet-dependent, and host-immune-dependent nature of the individual microbiome. The clinical application of FMT requires optimized donor selection criteria, standardized preparation protocols, appropriate delivery route selection, and long-term safety monitoring.

The interplay between the gut microbiota and cancer therapies is complex, with the microbiota influencing the efficacy and toxicity of treatments such as chemotherapy and immunotherapy [115,116,117]. Gut microbiota composition, specifically the abundance of *Akkermansia muciniphila* and *Ruminococcaceae*/*Faecalibacterium*, is associated with improved responses to PD-1/PD-L1 checkpoint inhibitors in epithelial tumors and melanoma patients, respectively [115,116]. In addition, certain bacterial increases irinotecan-induced intestinal mucositis through β-glucuronidase [118]. The summary of key in vivo studies supporting the gut–adipose–tumor axis is shown in Table 2. By modulating the gut microbiota, these therapies can potentially enhance therapeutic effects and reduce adverse effects, offering a novel avenue for improving cancer treatment.

While the therapeutic strategies described above hold scientific promise, it is essential to maintain a critical perspective on the current evidence base for gut microbiome recovery. Microbial composition varies substantially across ethnicities, geographies, ages, and diets. This individual variability severely limits the generalizability of microbiome-targeting interventions. The resilience and stability of the obese gut microbiome present a substantial biological barrier to durable remodeling. The microbiome exists in a dynamic equilibrium shaped by host genetics, chronic dietary patterns, intestinal immune tone, and the entrenched metabolic state of obesity itself. Short-term dietary interventions produce rapid but often transient shifts in microbiome composition. In addition, the risk-benefit profile of FMT in cancer therapy should be evaluated carefully.

## 6. Conclusions and Prospects

The gut–adipose–tumor axis represents a paradigm shift in our understanding of obesity-related carcinogenesis, revealing an intricate network of bidirectional communications. Key mechanisms and molecular mediators of the gut–adipose–tumor axis in obesity-related cancer are shown in Table 1. The axis reveals how metabolic disease can reshape distant tissues to favor malignant transformation. The gut microbiome emerges as a critical orchestrator in this tripartite relationship, with obesity-related dysbiosis triggering a cascade of pathological events. The shift toward pro-inflammatory bacterial communities, coupled with compromised intestinal barrier integrity, facilitates the systemic dissemination of microbial products that profoundly influence both adipose tissue homeostasis and distant tumor development. The reduction in beneficial SCFA-producing bacteria, alterations in bile acid metabolism, and increased production of genotoxic metabolites collectively create a systemic carcinogenic environment in the host.

The chronic low-grade inflammation characteristic of obese adipose tissue, marked by macrophage infiltration and pro-inflammatory cytokine production, establishes a systemic inflammatory state. The dramatic shifts in adipokine secretion, particularly elevated leptin and reduced adiponectin, create hormonal imbalances that directly stimulate cancer cell proliferation, survival, and invasion. Furthermore, the dysfunction of adipose tissue leads to metabolic disorders, including insulin resistance, hyperinsulinemia, and altered lipid metabolism, which provide growth signals and metabolic substrates necessary for the proliferation of tumor cells.

The tumor microenvironment in obesity is influenced by the dual signals derived from the gut and adipose. The altered immune landscape, characterized by immunosuppressive cell populations and impaired anti-tumor immunity, reflects the systemic immunometabolic disruption originating from gut dysbiosis and adipose inflammation. This axis operates through mechanisms including the translocation of specific bacterial species, systemic inflammation, circulating metabolites and hormones, and immune cell trafficking that collectively establish a tumorigenic systemic environment. The relative contributions of these mechanisms vary across cancer types.

The recognition of the gut–adipose–tumor axis as an integrated system opens unprecedented therapeutic opportunities. Rather than targeting individual components in isolation, interventions that simultaneously modulate multiple nodes within this network hold particular promise. Lifestyle modifications, including dietary interventions and physical activity, exert pleiotropic effects, such as reducing inflammation, restoring microbial diversity, improving adipose tissue function, and enhancing anti-tumor immunity. Emerging microbiome-targeted therapies, from probiotics to engineered bacteria, offer strategies to rebuild the gut ecosystem. Pharmacological agents such as metformin and GLP-1 receptor agonists demonstrate the potential to simultaneously improve metabolic health and exhibit anti-cancer properties. The integration of these approaches with conventional cancer therapies, particularly immunotherapy, may enhance treatment efficacy. Personalized medical strategies should be conducted to account for individual differences in microbiome composition, adipose tissue distribution, and metabolic phenotypes.

Most studies have been conducted in preclinical models, and rigorous validation is needed in different cancer populations. A meta-analysis including 33 cohort studies demonstrates that bariatric surgery reduces overall cancer risk (OR = 0.56; 95% CI = 0.46–0.68) [119]. The cancer-protective effects of bariatric surgery are partially attributed to modulation of gut microbiota composition, reduction in systemic inflammation, and normalization of metabolic hormones. Clinical evidences support that the use of metformin (adjusted RR for CRC = 0.884, 95% CI = 0.829–0.943) and GLP-1 receptor agonists (liraglutide and semaglutide) reduces cancer risk in diabetic/obese patients [78,120]. In terms of diet, a clinical trial demonstrates that a high-fiber diet improves outcomes in cancer patients receiving immunotherapy, providing direct human evidence that gut microbiota modulation via diet enhances anti-tumor immunity [121]. Phase I/II clinical trials have demonstrated that FMT from healthy donors improves gut microbiota diversity and metabolic parameters in obese patients [122]. FMT from immunotherapy-responding donors has been tested in phase I trials for melanoma, with preliminary evidence of restored anti-tumor immune responses in checkpoint inhibitor-refractory patients [123]. Studies employing multi-omics integration are essential to explore the dynamic evolution of the gut–adipose–tumor axis during obesity development and cancer progression. Advanced experimental models, including humanized gnotobiotic mice, adipose–tumor co-culture systems, and patient-derived organoids, will provide mechanistic insights while maintaining human relevance. The identification and validation of axis-specific biomarkers that predict cancer risk, prognosis, and therapeutic response could enable risk stratification and individualized treatment. In addition, clinical trials should be specifically designed to assess interventions targeting the gut–adipose–tumor axis. Given that obesity is a modifiable risk factor, interventions that target the axis represent potential strategies for reducing the global burden of obesity-related cancers.

## Figures and Tables

**Table 1:** Key Mechanisms and molecular mediators of the gut–adipose–tumor axis in obesity-related cancer.

Axis Node	Mechanism	Key Mediators/Molecules	Cancer-Promoting Effect
Gut Microbiota	Dysbiosis/reduced diversity	↓ *Firmicutes*/*Bacteroidetes ratio*; ↓ *Bifidobacterium*, *Lactobacillus*; ↑ *Fusobacterium nucleatum*, *Proteobacteria*	Chronic inflammation; immune dysregulation; barrier impairment
Gut Microbiota	Metabolic endotoxemia	LPS (from Gram-negative bacteria) → TLR4/NF-κB activation	Systemic inflammation; insulin resistance; tumor promotion
Gut Microbiota	↓ SCFA production	↓ Butyrate, propionate, acetate (from ↓ *Bacteroidetes*/*Firmicutes*)	Loss of HDAC inhibition; ↓ apoptosis; ↑ colorectal cancer risk
Gut Microbiota	Secondary bile acid excess	DCA, LCA (from *Clostridium scindens*-mediated 7α-dehydroxylation)	DNA damage; EMT induction; hepatic stellate cell activation (HCC)
Gut Microbiota	Carcinogenic metabolite production	TMAO (from choline/carnitine); *N*-nitroso compounds; H_2_S	Genotoxicity; endothelial dysfunction; CRC promotion
Gut Microbiota	Estrogen dysregulation	β-glucuronidase (*Clostridiales*) → deconjugation of estrogen metabolites	↑ Circulating estrogens → postmenopausal breast cancer risk
Adipose Tissue	Chronic low-grade inflammation	TNF-α, IL-6, IL-1β, MCP-1 (from hypertrophic adipocytes + M1 macrophages)	NF-κB/STAT3 activation; tumor proliferation; angiogenesis
Adipose Tissue	Adipokine imbalance	↑Leptin (JAK2/STAT3, MAPK, PI3K/AKT) ↓adiponectin (AMPK activation)	↑ Cell proliferation; ↓ apoptosis; ↑ VEGF; ↑ MMP-9
Adipose Tissue	Hyperinsulinemia/IGF-1 axis	↑ Insulin → ↓ IGFBP-1/2 → ↑ free IGF-1 → IGF-1R/PI3K/AKT/mTOR	↑ Tumor anabolism; ↑ cell survival; ↓ apoptosis
Adipose Tissue	NLRP3 inflammasome activation	IL-1β, IL-18 (via mitochondrial dysfunction and ROS)	Insulin resistance; macrophage M1 polarization; systemic inflammation
Adipose Tissue	Exosome-mediated crosstalk	Adipose-derived exosomes carrying miR-155, miR-23a, fatty acids	EMT; metastasis; metabolic reprogramming in tumor cells
Tumor Microenvironment	Immunosuppression	↑ Tregs, ↑ MDSCs, ↓ CD8+ T cells, ↑ PD-L1 expression	Impaired anti-tumor immunity; immune checkpoint resistance
Tumor Microenvironment	Angiogenesis	↑ VEGF (via HIF-1α, STAT3, NF-κB)	Tumor vascularization; ↑ metastatic potential
Tumor Microenvironment	Epithelial–mesenchymal transition	↓ E-cadherin; ↑ vimentin, fibronectin, N-cadherin; ↑ Snail, Twist, ZEB1	Invasion; metastasis; cancer stem cell generation
Gut → Adipose Crosstalk	LPS-driven adipose inflammation	LPS → TLR4 on adipocytes/macrophages → NF-κB → TNF-α, IL-6 secretion	Adipose dysfunction; systemic insulin resistance
Adipose → Gut Crosstalk	Barrier integrity compromise	Adipose-derived TNF-α, IL-6 → ↓ ZO-1, claudin-1 expression in enterocytes	↑ Intestinal permeability; ↑ microbial translocation; amplified dysbiosis

DCA: deoxycholic acid; LCA: lithocholic acid; LPS: lipopolysaccharide; SCFAs: short-chain fatty acids; TMAO: trimethylamine N-oxide; EMT: epithelial–mesenchymal transition; HCC: hepatocellular carcinoma; CRC: colorectal cancer; IGF-1R: insulin-like growth factor-1 receptor; HDAC: histone deacetylase; MDSCs: myeloid-derived suppressor cells; Tregs: regulatory T cells; VEGF: vascular endothelial growth factor; MMP: matrix metalloproteinase. ↑: increased, ↓: reduced.

**Table 2:** Summary of key in vivo studies supporting the gut–adipose–tumor axis.

Intervention/Model	Animal Model	Key Findings	Section
Klebsiella aerogenes colonization	Mouse (CRC)	↑ Colonic tumor number/size; ↑ TNF-α, IL-1β	Section 4.1
Fusobacterium nucleatum inoculation	Mouse (CRC)	↑ Tumor proliferation via TLR4/NF-κB; ↑ IL-6	Section 4.1
Antibiotic treatment	Mouse (HCC)	Reduced hepatic tumor burden	Section 4.1
High-fat diet (HFD) feeding	Mouse (breast cancer)	↑ Mammary adipose inflammation; ↑ CLS density correlated with tumor size	Section 4.2
FMT from HFD-fed donors	Mouse (breast cancer)	↑ Mammary tumor onset and metastasis	Section 4.3
FMT from HFD-fed donors	Germ-free mice	More adipose mass	Section 4.3
Metformin treatment (obese/T2D model)	Mouse	Inhibited mTOR pathway; reduced colorectal tumor incidence (RR = 0.884)	Section 5.1
GLP-1 agonist (liraglutide)	Mouse/clinical	Reduced body weight, insulin resistance, and cancer risk in T2D patients	Section 5.1
Exercise intervention (aerobic)	Mouse/human	↓ Adipose inflammation; ↓ macrophage infiltration; improved FGF21 signaling	Section 5.2
FMT from healthy donors	Mouse (CRC)	Normalized gut microbiota; reduced tumor growth	Section 5.3
Galacto-oligosaccharides from lactulose supplementation	Mouse (CRC)	Significantly reduced number of colonic tumors	Section 5.3
Lactobacillus rhamnosus administration	Mouse/rat	Reduced pro-inflammatory taxa; ↑ SCFA production; anti-carcinogenic effects	Section 5.3

CRC: colorectal cancer; CLSs: crown-like structures; SCFAs: short-chain fatty acids; FMT: fecal microbiota transplantation; T2D: type 2 diabetes; and FGF21: fibroblast growth factor 21. ↑: increased, ↓: reduced.

## Data Availability

No new data were created or analyzed in this study.

## References

[1] Avgerinos K.I., Spyrou N., Mantzoros C.S., Dalamaga M. (2019). Obesity and cancer risk: Emerging biological mechanisms and perspectives. Metabolism.

[2] Gujarathi R., Klein J.A., Liao C.Y., Pillai A. (2025). The Changing Demographics and Epidemiology of Hepatocellular Carcinoma. Clin. Liver Dis..

[3] McGlynn K.A., Petrick J.L., El-Serag H.B. (2021). Epidemiology of Hepatocellular Carcinoma. Hepatology.

[4] Ruiz-Malagon A.J., Rodriguez-Sojo M.J., Redondo E., Rodriguez-Cabezas M.E., Galvez J., Rodriguez-Nogales A. (2025). Systematic review: The gut microbiota as a link between colorectal cancer and obesity. Obes. Rev..

[5] Hopkins B.D., Goncalves M.D., Cantley L.C. (2016). Obesity and Cancer Mechanisms: Cancer Metabolism. J. Clin. Oncol..

[6] Gomes A.C., Hoffmann C., Mota J.F. (2018). The human gut microbiota: Metabolism and perspective in obesity. Gut Microbes.

[7] Gallagher E.J., LeRoith D. (2020). Hyperinsulinaemia in cancer. Nat. Rev. Cancer.

[8] Yoshimoto S., Loo T.M., Atarashi K., Kanda H., Sato S., Oyadomari S., Iwakura Y., Oshima K., Morita H., Hattori M. (2013). Obesity-induced gut microbial metabolite promotes liver cancer through senescence secretome. Nature.

[9] Song X., An Y., Chen D., Zhang W., Wu X., Li C., Wang S., Dong W., Wang B., Liu T. (2022). Microbial metabolite deoxycholic acid promotes vasculogenic mimicry formation in intestinal carcinogenesis. Cancer Sci..

[10] Day-Walsh P., Shehata E., Saha S., Savva G.M., Nemeckova B., Speranza J., Kellingray L., Narbad A., Kroon P.A. (2021). The use of an in-vitro batch fermentation (human colon) model for investigating mechanisms of TMA production from choline, L-carnitine and related precursors by the human gut microbiota. Eur. J. Nutr..

[11] Saaoud F., Liu L., Xu K., Cueto R., Shao Y., Lu Y., Sun Y., Snyder N.W., Wu S., Yang L. (2023). Aorta- and liver-generated TMAO enhances trained immunity for increased inflammation via ER stress/mitochondrial ROS/glycolysis pathways. JCI Insight.

[12] Yan Y., Li J., Xu H., Yang C., Li G., Feng Y., Wang Q., Li L., Wu R., Sun H. (2025). The food-derived metabolite trimethylamine and trimethylamine-N-oxide promote colorectal cancer progression via SREBF1. Ecotoxicol. Environ. Saf..

[13] Duan M., Wang Y., Zhang Q., Zou R., Guo M., Zheng H. (2021). Characteristics of gut microbiota in people with obesity. PLoS ONE.

[14] Gao R., Zhu C., Li H., Yin M., Pan C., Huang L., Kong C., Wang X., Zhang Y., Qu S. (2018). Dysbiosis Signatures of Gut Microbiota Along the Sequence from Healthy, Young Patients to Those with Overweight and Obesity. Obesity.

[15] Amabebe E., Robert F.O., Agbalalah T., Orubu E.S.F. (2020). Microbial dysbiosis-induced obesity: Role of gut microbiota in homoeostasis of energy metabolism. Br. J. Nutr..

[16] Nagpal R., Newman T.M., Wang S., Jain S., Lovato J.F., Yadav H. (2018). Obesity-Linked Gut Microbiome Dysbiosis Associated with Derangements in Gut Permeability and Intestinal Cellular Homeostasis Independent of Diet. J. Diabetes Res..

[17] Liu R., Hong J., Xu X., Feng Q., Zhang D., Gu Y., Shi J., Zhao S., Liu W., Wang X. (2017). Gut microbiome and serum metabolome alterations in obesity and after weight-loss intervention. Nat. Med..

[18] Crovesy L., Masterson D., Rosado E.L. (2020). Profile of the gut microbiota of adults with obesity: A systematic review. Eur. J. Clin. Nutr..

[19] Samuel B.S., Gordon J.I. (2006). A humanized gnotobiotic mouse model of host-archaeal-bacterial mutualism. Proc. Natl. Acad. Sci. USA.

[20] Feng X., Deng M., Zhang L., Pan Q. (2023). Impact of gut microbiota and associated mechanisms on postprandial glucose levels in patients with diabetes. J. Transl. Intern. Med..

[21] Zhong Y., Lei Y., Jiang S., Chen D., Wang X., Wang K., Liao T., Liao R., Gan M., Niu L. (2025). Advances in understanding the role of gut microbiota in fat deposition and lipid metabolism. J. Anim. Sci. Biotechnol..

[22] Shukla P.K., Meena A.S., Manda B., Gomes-Solecki M., Dietrich P., Dragatsis I., Rao R. (2018). Lactobacillus plantarum prevents and mitigates alcohol-induced disruption of colonic epithelial tight junctions, endotoxemia, and liver damage by an EGF receptor-dependent mechanism. FASEB J..

[23] Vlasova A.N., Kandasamy S., Chattha K.S., Rajashekara G., Saif L.J. (2016). Comparison of probiotic lactobacilli and bifidobacteria effects, immune responses and rotavirus vaccines and infection in different host species. Vet. Immunol. Immunopathol..

[24] Mabrok H.B., Ramadan A.A., Hamed I.M., Mohamed D.A. (2024). Obesity as Inducer of Cognitive Function Decline via Dysbiosis of Gut Microbiota in Rats. Brain Sci..

[25] May K.S., den Hartigh L.J. (2021). Modulation of Adipocyte Metabolism by Microbial Short-Chain Fatty Acids. Nutrients.

[26] Iqbal M., Yu Q., Tang J., Xiang J. (2025). Unraveling the gut microbiota’s role in obesity: Key metabolites, microbial species, and therapeutic insights. J. Bacteriol..

[27] de la Cuesta-Zuluaga J., Mueller N.T., Alvarez-Quintero R., Velasquez-Mejia E.P., Sierra J.A., Corrales-Agudelo V., Carmona J.A., Abad J.M., Escobar J.S. (2018). Higher Fecal Short-Chain Fatty Acid Levels Are Associated with Gut Microbiome Dysbiosis, Obesity, Hypertension and Cardiometabolic Disease Risk Factors. Nutrients.

[28] Huang D., Xiong M., Xu X., Wu X., Xu J., Cai X., Lu L., Zhou H. (2020). Bile acids elevated by high-fat feeding induce endoplasmic reticulum stress in intestinal stem cells and contribute to mucosal barrier damage. Biochem. Biophys. Res. Commun..

[29] Jumpertz R., Le D.S., Turnbaugh P.J., Trinidad C., Bogardus C., Gordon J.I., Krakoff J. (2011). Energy-balance studies reveal associations between gut microbes, caloric load, and nutrient absorption in humans. Am. J. Clin. Nutr..

[30] Everard A., Cani P.D. (2014). Gut microbiota and GLP-1. Rev. Endocr. Metab. Disord..

[31] Duca F.A., Sakar Y., Covasa M. (2013). The modulatory role of high fat feeding on gastrointestinal signals in obesity. J. Nutr. Biochem..

[32] Bluher M. (2013). Adipose tissue dysfunction contributes to obesity related metabolic diseases. Best Pract. Res. Clin. Endocrinol. Metab..

[33] Xu L., Yan X., Zhao Y., Wang J., Liu B., Yu S., Fu J., Liu Y., Su J. (2022). Macrophage Polarization Mediated by Mitochondrial Dysfunction Induces Adipose Tissue Inflammation in Obesity. Int. J. Mol. Sci..

[34] Longo M., Zatterale F., Naderi J., Parrillo L., Formisano P., Raciti G.A., Beguinot F., Miele C. (2019). Adipose Tissue Dysfunction as Determinant of Obesity-Associated Metabolic Complications. Int. J. Mol. Sci..

[35] Kawai T., Autieri M.V., Scalia R. (2021). Adipose tissue inflammation and metabolic dysfunction in obesity. Am. J. Physiol. Cell Physiol..

[36] Fernandez-Alfonso M.S., Gil-Ortega M., Garcia-Prieto C.F., Aranguez I., Ruiz-Gayo M., Somoza B. (2013). Mechanisms of perivascular adipose tissue dysfunction in obesity. Int. J. Endocrinol..

[37] Stanek A., Brozyna-Tkaczyk K., Myslinski W. (2021). The Role of Obesity-Induced Perivascular Adipose Tissue (PVAT) Dysfunction in Vascular Homeostasis. Nutrients.

[38] Trayhurn P. (2013). Hypoxia and adipose tissue function and dysfunction in obesity. Physiol. Rev..

[39] Nguyen L., Shanmugan S. (2024). A Review Article: The Relationship Between Obesity and Colorectal Cancer. Curr. Diabetes Rep..

[40] Mao H., Feng X.Z., Guang S.H. (2013). Treating liver cancer with antibiotics?. Acta Pharmacol. Sin..

[41] Yang Y., Weng W., Peng J., Hong L., Yang L., Toiyama Y., Gao R., Liu M., Yin M., Pan C. (2017). Fusobacterium nucleatum Increases Proliferation of Colorectal Cancer Cells and Tumor Development in Mice by Activating Toll-Like Receptor 4 Signaling to Nuclear Factor-kappaB, and Up-regulating Expression of MicroRNA-21. Gastroenterology.

[42] Han J., Zhang B., Zhang Y., Yin T., Cui Y., Liu J., Yang Y., Song H., Shang D. (2023). Gut microbiome: Decision-makers in the microenvironment of colorectal cancer. Front. Cell. Infect. Microbiol..

[43] Wang X., Meng M., Sun J., Gao W., Lin C., Yu C. (2024). Klebsiella aerogenes exacerbates colon tumorigenesis in the AOM/DSS-induced C57BL/6J mouse. Biochem. Biophys. Res. Commun..

[44] Feitelson M.A., Arzumanyan A., Medhat A., Spector I. (2023). Short-chain fatty acids in cancer pathogenesis. Cancer Metastasis Rev..

[45] Wong C.C., Yu J. (2023). Gut microbiota in colorectal cancer development and therapy. Nat. Rev. Clin. Oncol..

[46] Veettil S.K., Wong T.Y., Loo Y.S., Playdon M.C., Lai N.M., Giovannucci E.L., Chaiyakunapruk N. (2021). Role of Diet in Colorectal Cancer Incidence: Umbrella Review of Meta-analyses of Prospective Observational Studies. JAMA Netw. Open.

[47] Janney A., Powrie F., Mann E.H. (2020). Host-microbiota maladaptation in colorectal cancer. Nature.

[48] Jia W., Rajani C., Xu H., Zheng X. (2021). Gut microbiota alterations are distinct for primary colorectal cancer and hepatocellular carcinoma. Protein Cell.

[49] Guo X., Shao Y. (2025). Role of the oral-gut microbiota axis in pancreatic cancer: A new perspective on tumor pathophysiology, diagnosis, and treatment. Mol. Med..

[50] Flores R., Shi J., Fuhrman B., Xu X., Veenstra T.D., Gail M.H., Gajer P., Ravel J., Goedert J.J. (2012). Fecal microbial determinants of fecal and systemic estrogens and estrogen metabolites: A cross-sectional study. J. Transl. Med..

[51] Kisar Tunca S., Unal R. (2024). Adipocyte-derived fatty acid uptake induces obesity-related breast cancer progression: A review. Mol. Biol. Rep..

[52] Quail D.F., Dannenberg A.J. (2019). The obese adipose tissue microenvironment in cancer development and progression. Nat. Rev. Endocrinol..

[53] Cinti S., Mitchell G., Barbatelli G., Murano I., Ceresi E., Faloia E., Wang S., Fortier M., Greenberg A.S., Obin M.S. (2005). Adipocyte death defines macrophage localization and function in adipose tissue of obese mice and humans. J. Lipid Res..

[54] Goncalves R.M., Delgobo M., Agnes J.P., das Neves R.N., Falchetti M., Casagrande T., Garcia A.P.V., Vieira T.C., Somensi N., Bruxel M.A. (2021). COX-2 promotes mammary adipose tissue inflammation, local estrogen biosynthesis, and carcinogenesis in high-sugar/fat diet treated mice. Cancer Lett..

[55] Shen L., Zhang C., Cui K., Liang X., Zhu G., Hong L. (2024). Leptin secreted by adipocytes promotes EMT transition and endometrial cancer progression via the JAK2/STAT3 signalling pathway. Adipocyte.

[56] Guo S., Liu M., Wang G., Torroella-Kouri M., Gonzalez-Perez R.R. (2012). Oncogenic role and therapeutic target of leptin signaling in breast cancer and cancer stem cells. Biochim. Biophys. Acta.

[57] Nishida A., Andoh A. (2025). The Role of Inflammation in Cancer: Mechanisms of Tumor Initiation, Progression, and Metastasis. Cells.

[58] Moutabian H., Radi U.K., Saleman A.Y., Adil M., Zabibah R.S., Chaitanya M.N.L., Saadh M.J., Jawad M.J., Hazrati E., Bagheri H. (2023). MicroRNA-155 and cancer metastasis: Regulation of invasion, migration, and epithelial-to-mesenchymal transition. Pathol. Res. Pract..

[59] Vallianou N., Stratigou T., Christodoulatos G.S., Dalamaga M. (2019). Understanding the Role of the Gut Microbiome and Microbial Metabolites in Obesity and Obesity-Associated Metabolic Disorders: Current Evidence and Perspectives. Curr. Obes. Rep..

[60] Mamun M.A.A., Rakib A., Mandal M., Singh U.P. (2025). Impact of a High-Fat Diet on the Gut Microbiome: A Comprehensive Study of Microbial and Metabolite Shifts During Obesity. Cells.

[61] Cani P.D., Jordan B.F. (2018). Gut microbiota-mediated inflammation in obesity: A link with gastrointestinal cancer. Nat. Rev. Gastroenterol. Hepatol..

[62] Delzenne N.M., Neyrinck A.M., Backhed F., Cani P.D. (2011). Targeting gut microbiota in obesity: Effects of prebiotics and probiotics. Nat. Rev. Endocrinol..

[63] Rubinstein M.R., Wang X., Liu W., Hao Y., Cai G., Han Y.W. (2013). Fusobacterium nucleatum promotes colorectal carcinogenesis by modulating E-cadherin/beta-catenin signaling via its FadA adhesin. Cell Host Microbe.

[64] Benvenuti L., D’Antongiovanni V., Pellegrini C., Fornai M., Bernardini N., Ippolito C., Segnani C., Di Salvo C., Colucci R., Martelli A. (2023). Dietary Supplementation with the Probiotic SF68 Reinforces Intestinal Epithelial Barrier in Obese Mice by Improving Butyrate Bioavailability. Mol. Nutr. Food Res..

[65] Chen J., Liu X., Zou Y., Gong J., Ge Z., Lin X., Zhang W., Huang H., Zhao J., Saw P.E. (2024). A high-fat diet promotes cancer progression by inducing gut microbiota-mediated leucine production and PMN-MDSC differentiation. Proc. Natl. Acad. Sci. USA.

[66] Ridaura V.K., Faith J.J., Rey F.E., Cheng J., Duncan A.E., Kau A.L., Griffin N.W., Lombard V., Henrissat B., Bain J.R. (2013). Gut microbiota from twins discordant for obesity modulate metabolism in mice. Science.

[67] Hersoug L.G., Moller P., Loft S. (2016). Gut microbiota-derived lipopolysaccharide uptake and trafficking to adipose tissue: Implications for inflammation and obesity. Obes. Rev..

[68] Cani P.D., Van Hul M. (2024). Gut microbiota in overweight and obesity: Crosstalk with adipose tissue. Nat. Rev. Gastroenterol. Hepatol..

[69] Fang W., Wang K., Wen S., Zhou F., Ouyang J., Zhang S., Zeng H., Hara Y., Huang J.A., Liu Z. (2025). Theaflavins in black tea ameliorate high-fat diet-induced obesity and inflammation via gut microbiota, AMPK-mediated metabolism, and NF-kappaB pathway. Phytomedicine.

[70] Pi-Sunyer X., Astrup A., Fujioka K., Greenway F., Halpern A., Krempf M., Lau D.C., le Roux C.W., Violante Ortiz R., Jensen C.B. (2015). A Randomized, Controlled Trial of 3.0 mg of Liraglutide in Weight Management. N. Engl. J. Med..

[71] Drucker D.J. (2018). Mechanisms of Action and Therapeutic Application of Glucagon-like Peptide-1. Cell Metab..

[72] Wang L., Xu R., Kaelber D.C., Berger N.A. (2024). Glucagon-Like Peptide 1 Receptor Agonists and 13 Obesity-Associated Cancers in Patients With Type 2 Diabetes. JAMA Netw. Open.

[73] Kanbay M., Al-Shiab R., Shah E., Ozbek L., Guldan M., Ortiz A., Fouque D. (2025). Gut microbiota modulation in GLP-1RA and SGLT-2i therapy: Clinical implications and mechanistic insights in type 2 diabetes. Clin. Kidney J..

[74] Helmstadter J., Keppeler K., Aust F., Kuster L., Frenis K., Filippou K., Vujacic-Mirski K., Tsohataridis S., Kalinovic S., Kroller-Schon S. (2021). GLP-1 Analog Liraglutide Improves Vascular Function in Polymicrobial Sepsis by Reduction of Oxidative Stress and Inflammation. Antioxidants.

[75] Mallik R., Chowdhury T.A. (2018). Metformin in cancer. Diabetes Res. Clin. Pract..

[76] de la Cuesta-Zuluaga J., Mueller N.T., Corrales-Agudelo V., Velasquez-Mejia E.P., Carmona J.A., Abad J.M., Escobar J.S. (2017). Metformin Is Associated With Higher Relative Abundance of Mucin-Degrading Akkermansia muciniphila and Several Short-Chain Fatty Acid-Producing Microbiota in the Gut. Diabetes Care.

[77] Wei X.L., Tao M.H., Li R.H., Ge S.H., Xiao W. (2025). Metformin and Adipose Tissue: A Multifaceted Regulator in Metabolism, Inflammation, and Regeneration. Endocrinol. Metab..

[78] Yang W.T., Yang H.J., Zhou J.G., Liu J.L. (2020). Relationship between metformin therapy and risk of colorectal cancer in patients with diabetes mellitus: A meta-analysis. Int. J. Color. Dis..

[79] Biondo L.A., Teixeira A.A.S., Ferreira K.C.d.O.S., Neto J.C.R. (2020). Pharmacological Strategies for Insulin Sensitivity in Obesity and Cancer: Thiazolidinediones and Metformin. Curr. Pharm. Des..

[80] Rennert G., Rennert H.S., Gronich N., Pinchev M., Gruber S.B. (2020). Use of metformin and risk of breast and colorectal cancer. Diabetes Res. Clin. Pract..

[81] Zhang S., Ren X., Zhang B., Lan T., Liu B. (2024). A Systematic Review of Statins for the Treatment of Nonalcoholic Steatohepatitis: Safety, Efficacy, and Mechanism of Action. Molecules.

[82] Kauerova S., Bartuskova H., Muffova B., Janousek L., Fronek J., Petras M., Poledne R., Kralova Lesna I. (2021). Statins Directly Influence the Polarization of Adipose Tissue Macrophages: A Role in Chronic Inflammation. Biomedicines.

[83] Nyboe Andersen N., Wildt S., Iversen A.T., Poulsen G., Jess T., Munck L.K., Borup C. (2024). Risk of cancer in patients with bile acid diarrhoea: A Danish nationwide matched cohort study. BMJ Open Gastroenterol..

[84] Zhao H., Yang F., Yang J., Yang S. (2025). Multifaceted roles of microbiota-derived deoxycholic acid in gastrointestinal cancers: From barrier disruption to therapeutic implications. Hum. Cell.

[85] Farhana L., Nangia-Makker P., Arbit E., Shango K., Sarkar S., Mahmud H., Hadden T., Yu Y., Majumdar A.P. (2016). Bile acid: A potential inducer of colon cancer stem cells. Stem Cell Res. Ther..

[86] Silva Y.P., Bernardi A., Frozza R.L. (2020). The Role of Short-Chain Fatty Acids From Gut Microbiota in Gut-Brain Communication. Front. Endocrinol..

[87] Makki K., Deehan E.C., Walter J., Backhed F. (2018). The Impact of Dietary Fiber on Gut Microbiota in Host Health and Disease. Cell Host Microbe.

[88] Fernandez E., Wargo J.A., Helmink B.A. (2025). The Microbiome and Cancer: A Translational Science Review. JAMA.

[89] Sudhakaran M., Doseff A.I. (2020). The Targeted Impact of Flavones on Obesity-Induced Inflammation and the Potential Synergistic Role in Cancer and the Gut Microbiota. Molecules.

[90] Limijadi E.K.S., Tjandra K.C., Permatasari H.K., Augusta P.S., Surya R., Harbuwono D.S., Nurkolis F. (2025). Marine-Algal-Derived Postbiotics Modulating the Gut Microbiota-Adipose Tissue Axis in Obesity: A New Frontier. Nutrients.

[91] Jun J.K., Lee W.L., Park H.G., Lee S.K., Jeong S.H., Lee Y.R. (2014). Moderate intensity exercise inhibits macrophage infiltration and attenuates adipocyte inflammation in ovariectomized rats. J. Exerc. Nutr. Biochem..

[92] Rosa-Neto J.C., Silveira L.S. (2020). Endurance Exercise Mitigates Immunometabolic Adipose Tissue Disturbances in Cancer and Obesity. Int. J. Mol. Sci..

[93] Geng L., Liao B., Jin L., Huang Z., Triggle C.R., Ding H., Zhang J., Huang Y., Lin Z., Xu A. (2019). Exercise Alleviates Obesity-Induced Metabolic Dysfunction via Enhancing FGF21 Sensitivity in Adipose Tissues. Cell Rep..

[94] Della Guardia L., Codella R. (2023). Exercise Restores Hypothalamic Health in Obesity by Reshaping the Inflammatory Network. Antioxidants.

[95] McTiernan A., Friedenreich C.M., Katzmarzyk P.T., Powell K.E., Macko R., Buchner D., Pescatello L.S., Bloodgood B., Tennant B., Vaux-Bjerke A. (2019). Physical Activity in Cancer Prevention and Survival: A Systematic Review. Med. Sci. Sports Exerc..

[96] Kim M.S., Kim J.Y., Song Y.S., Hong S., Won H.H., Kim W.J., Kwon Y., Ha J., Fiedorowicz J.G., Solmi M. (2024). Association of bariatric surgery with indicated and unintended outcomes: An umbrella review and meta-analysis for risk-benefit assessment. Obes. Rev..

[97] Patel R., DuPont H.L. (2015). New approaches for bacteriotherapy: Prebiotics, new-generation probiotics, and synbiotics. Clin. Infect. Dis..

[98] Panebianco C., Latiano T., Pazienza V. (2020). Microbiota Manipulation by Probiotics Administration as Emerging Tool in Cancer Prevention and Therapy. Front. Oncol..

[99] Jiang S., Ma W., Ma C., Zhang Z., Zhang W., Zhang J. (2024). An emerging strategy: Probiotics enhance the effectiveness of tumor immunotherapy via mediating the gut microbiome. Gut Microbes.

[100] Rahmani N., Pourali G., Hosseini N., Fiuji H., Maftooh M., Hassanian S.M., Ferns G.A., Khazaei M., Avan A. (2023). Probiotics as a Therapeutic Approach in Colorectal Cancer. Curr. Cancer Drug Targets.

[101] Ahn K., Baek K.W., Yun K., Oh Y., Kim Y.S., Im E., Lee Y., Choi J., Song E.J., Park Y.S. (2025). The effects of candidate probiotic strains on the gut environment in dextran sulfate sodium-induced colitis mouse. Sci. Rep..

[102] Delzenne N.M., Bindels L.B., Neyrinck A.M., Walter J. (2025). The gut microbiome and dietary fibres: Implications in obesity, cardiometabolic diseases and cancer. Nat. Rev. Microbiol..

[103] Barchelouei N.K., Yazdi M.H., Haghighat S. (2025). Probiotic intervention alters immune gene expression and tumor characteristics in experimental breast cancer. Mol. Biol. Rep..

[104] Parnell J.A., Reimer R.A. (2012). Prebiotic fiber modulation of the gut microbiota improves risk factors for obesity and the metabolic syndrome. Gut Microbes.

[105] Perillo F., Amoroso C., Strati F., Giuffre M.R., Diaz-Basabe A., Lattanzi G., Facciotti F. (2020). Gut Microbiota Manipulation as a Tool for Colorectal Cancer Management: Recent Advances in Its Use for Therapeutic Purposes. Int. J. Mol. Sci..

[106] Hijova E., Szabadosova V., Stofilova J., Hrckova G. (2013). Chemopreventive and metabolic effects of inulin on colon cancer development. J. Vet. Sci..

[107] Hijova E., Szabadosova V., Strojny L., Bomba A. (2014). Changes chemopreventive markers in colorectal cancer development after inulin supplementation. Bratisl. Lek. Listy.

[108] Li H.Y., Zhou D.D., Gan R.Y., Huang S.Y., Zhao C.N., Shang A., Xu X.Y., Li H.B. (2021). Effects and Mechanisms of Probiotics, Prebiotics, Synbiotics, and Postbiotics on Metabolic Diseases Targeting Gut Microbiota: A Narrative Review. Nutrients.

[109] Alam Z., Shang X., Effat K., Kanwal F., He X., Li Y., Xu C., Niu W., War A.R., Zhang Y. (2022). The potential role of prebiotics, probiotics, and synbiotics in adjuvant cancer therapy especially colorectal cancer. J. Food Biochem..

[110] Kvakova M., Kamlarova A., Stofilova J., Benetinova V., Bertkova I. (2022). Probiotics and postbiotics in colorectal cancer: Prevention and complementary therapy. World J. Gastroenterol..

[111] Zikou E., Koliaki C., Makrilakis K. (2024). The Role of Fecal Microbiota Transplantation (FMT) in the Management of Metabolic Diseases in Humans: A Narrative Review. Biomedicines.

[112] Napolitano M., Covasa M. (2020). Microbiota Transplant in the Treatment of Obesity and Diabetes: Current and Future Perspectives. Front. Microbiol..

[113] Baruch E.N., Youngster I., Ben-Betzalel G., Ortenberg R., Lahat A., Katz L., Adler K., Dick-Necula D., Raskin S., Bloch N. (2021). Fecal microbiota transplant promotes response in immunotherapy-refractory melanoma patients. Science.

[114] Davar D., Dzutsev A.K., McCulloch J.A., Rodrigues R.R., Chauvin J.M., Morrison R.M., Deblasio R.N., Menna C., Ding Q., Pagliano O. (2021). Fecal microbiota transplant overcomes resistance to anti-PD-1 therapy in melanoma patients. Science.

[115] Routy B., Le Chatelier E., Derosa L., Duong C.P.M., Alou M.T., Daillere R., Fluckiger A., Messaoudene M., Rauber C., Roberti M.P. (2018). Gut microbiome influences efficacy of PD-1-based immunotherapy against epithelial tumors. Science.

[116] Gopalakrishnan V., Spencer C.N., Nezi L., Reuben A., Andrews M.C., Karpinets T.V., Prieto P.A., Vicente D., Hoffman K., Wei S.C. (2018). Gut microbiome modulates response to anti-PD-1 immunotherapy in melanoma patients. Science.

[117] Li S., Zhu S., Yu J. (2024). The role of gut microbiota and metabolites in cancer chemotherapy. J. Adv. Res..

[118] Sadeghloo Z., Sadeghi A. (2025). Gut microbiota as a hidden modulator of chemotherapy: Implications for colorectal cancer treatment. Discov. Oncol..

[119] Chen Z.W., Jin T., Liang P.P., Li Z.D., He F.J., Chen Z.H., Song X.H., Zhu Y.F., Hu J.K., Yang K. (2024). Incidence of cancer for patients after bariatric surgery: Evidence from 33 cohort studies. Surg. Obes. Relat. Dis..

[120] Levy S., Attia A., Elshazli R.M., Abdelmaksoud A., Tatum D., Aiash H., Toraih E.A. (2024). Differential Effects of GLP-1 Receptor Agonists on Cancer Risk in Obesity: A Nationwide Analysis of 1.1 Million Patients. Cancers.

[121] Farias R.M., Jiang Y., Levy E.J., Hwang C., Wang J., Burton E.M., Cohen L., Ajami N., Wargo J.A., Daniel C.R. (2024). Diet and Immune Effects Trial (DIET)—A randomized, double-blinded dietary intervention study in patients with melanoma receiving immunotherapy. BMC Cancer.

[122] Zhang Z., Mocanu V., Deehan E.C., Hotte N., Zhu Y., Wei S., Kao D.H., Karmali S., Birch D.W., Walter J. (2024). Recipient microbiome-related features predicting metabolic improvement following fecal microbiota transplantation in adults with severe obesity and metabolic syndrome: A secondary analysis of a phase 2 clinical trial. Gut Microbes.

[123] Routy B., Lenehan J.G., Miller W.H., Jamal R., Messaoudene M., Daisley B.A., Hes C., Al K.F., Martinez-Gili L., Puncochar M. (2023). Fecal microbiota transplantation plus anti-PD-1 immunotherapy in advanced melanoma: A phase I trial. Nat. Med..

